# Determination of non-cholesterol sterols in serum and HDL fraction by LC/MS-MS: Significance of matrix-related interferences

**DOI:** 10.2478/jomb-2019-0044

**Published:** 2020-09-02

**Authors:** Sandra Vladimirov, Tamara Gojković, Aleksandra Zeljković, Zorana Jelić-Ivanović, Vesna Spasojević-Kalimanovska

**Affiliations:** 1 University of Belgrade, Faculty of Pharmacy, Department of Medical Biochemistry

**Keywords:** HPLC-MS/MS, cholesterol precursors, phytosterols, matrix effect, calibration, HPLC-MS/MS, prekursori holesterola, fitosteroli, efekat matriksa, kalibracija

## Abstract

**Background:**

Non-cholesterol sterols (NCS) are promising biomarkers for estimation of cholesterol homeostasis properties. In addition, determination of NCS in high-density lipoprotein (HDL) fraction (HDL-NCS) could provide information on cholesterol efflux. However, matrix effects interfere in liquid chromatography-mass spectrometry (LC-MS) analysis of NCS, thereby impairing the method sensitivity. The aims of this study were development, optimization and validation of LC-MS method for quantification of NCS in serum and HDL-NCS. Additionally, matrix effect interferences and methods application in individual serum samples were examined.

**Methods:**

HDL precipitating reagent was used for HDL isolation. Matrix effect was examined by comparing different surrogates by simple regression analysis. Validation was conducted according to the FDA-ICH guideline. 20 healthy volunteers were recruited for testing of method application.

**Results:**

The observed matrix effect was 30%, and matrix comparison showed that cholesterol was the dominant contributor to the matrix effect. Cholesterol concentration was adjusted by construction of the calibration curve for serum and HDL fraction (5 mmol/L and 2.5 mmol/L, respectively). The intraand interrun variabilities for NCSs were 4.7-10.3% for serum NCS and 3.6-13.6% for HDLNCS and 4.6-9.5% for serum NCSs and 2.5-9.8% for HDL-NCS, respectively. Recovery studies showed satisfactory results for NCSs: 89.8-113.1% for serum NCS and 85.3-95.8% for HDL-NCS.

**Conclusions:**

The method was successfully developed and optimized. The matrix interference was solved by customising calibration curves for each method and sample type. The measurement of NCS in HDL fraction was proposed for the first time as potentially useful procedure in biomedical researches.

## Abstract (sr)

**Uvod:**

Neholesterolski steroli (NHS) predstavljaju potencijalne biomarkere homeostaze holesterola. Pored toga, određivanje NHS u HDL frakciji (HDL-NHS) moglo bi da pruži dodatne informacije o efluksu holesterola. Međutim, određivanje NHS metodom tečne hromatografije - masene spektrometrije (LC-MS) je podložno uticaju matriksa usled čega dolazi do sniženja osetljivost metode. Studija je prvenstveno imala za cilj razvoj, optimizaciju i validaciju LC-MS metode za kvantifikaciju NHS u serumu i HDL-NHS. Pored toga, detaljno je ispitan efekat matriksa i metoda primenjena za analizu uzoraka seruma zdravih dobrovoljaca.

**Metode:**

Izolacija HDL frakcije je izvršena upotrebom precipitirajućeg reagensa. Efekat matriksa je ispitan poređenjem različitih surogat matriksa regresionom analizom. Validacija je sprovedena u skladu sa FDA-ICH vodičem za validaciju bioanalitičkih metoda. Korišćeni su serumi 20 zdravih dobrovoljaca u cilju kliničke evaluacije metode.

**Rezultati:**

Efekat matriksa je iznosio 30% i dalja analiza je pokazala da je holesterol bio glavni uzročnik efekta matriksa. U cilju rešavanja ove interferencije, kalibracione krive za su pripremljene u metanolnom rastvoru holesterola koncentracije 5 mmol/L, a za kvantifikaciju HDL-NHS u metanolnom rastvoru holesterola koncentracije 2,5 mmol/L. Varijacije unutar serije su bile 4,7-10,3% za NHS i 3,6-13,6% za HDL-NHS, a između serija 4,6-9,5% za NHS i 2,5-9,8% za HDL-NHS. Studije prinosa analita su pokazale zadovoljavajuće rezultate: 89,8-113,1% za NHS i 85,3-95,8% za HDL-NHS.

**Zaključak:**

Metoda je uspešno optimizovana i validirana. Interferencija usled prisustva holesterola je korigovana upotrebom odgovarajućih kalibracionih krivih za svaku vrstu uzorka. Određivanje HDL-NHS je po prvi put predloženo kao potencijalno korisna procedura u biomedicinskim istraživanjima.

## Introduction

Cholesterol metabolism is complexly regulated by cholesterol synthesis, absorption and elimination. Main contributors to cholesterol input are cholesterol synthesis and absorption processes, which are well balanced in healthy individuals, but dysregulated in many pathological states. Various diseases can induce an inability to maintain the cholesterol homeostasis and, thereby, lay the foundation for dyslipidaemiarelated comorbidities [1].

Non-cholesterol sterols (NCS) are regarded as reliable cholesterol synthesis and absorption surrogate markers. Most commonly analysed synthesis markers are lathosterol and desmosterol, while absorption can be monitored by quantification of phytosterols β-sitosterol and campesterol [2] [3]. Additionally, NCS analysis can be broaden to quantification of precursors common for multiple metabolic pathways such as 7-dehydrocholesterol (both cholesterol and vitamin D precursor) [4].

It is important to emphasize that the maintenance of cholesterol balance depends not only on the cholesterol synthesis and absorption patterns, but also on the distribution of cholesterol among various classes of lipoproteins [5]. HDL particle plays a significant role in cholesterol uptake from peripheral tissues and its transport to the liver, in the process called reverse cholesterol transport (RCT) [6]. This process is facilitated by ATP-binding transporters (ABC), proteins which allow the transport of a variety of substrates across lipid bilayers. Studies have shown that during the intensive cholesterol synthesis in extrahepatic cells, both cholesterol and its precursors (NCS) are being released onto HDL particle via ABCA1 and ABCG1 [7] [8]. Additionally, it is known that the intestine participates in cholesterol homeostasis since enterocytes control cholesterol absorption and biliary cholesterol elimination, but also plays a role in highdensity lipoprotein (HDL) biogenesis [6]. Since HDL maturation is closely related to the intestinal efflux of absorbed cholesterol to nascent HDL particles, it would be useful to explore the capacity of this process, by measuring cholesterol absorption markers solely in serum HDL fraction [7].

Therefore, by measuring cholesterol synthesis and absorption markers in the isolated HDL fraction, we could get a better insight into reverse cholesterol transport and overall cholesterol efflux from peripheral tissues. Having in mind that multiple protective properties of HDL particles strongly depends on their structure and protein/lipid levels [6], we could hypothesize that determination of NCSs in HDL fraction might be useful as potential indicator of HDL particles maturity and functionality. Such measurement could deliver a great clinical and research utility by allowing the assessment of RCT capacity in different types of dyslipidemia.

In the past, NCS bioanalysis methods by liquid chromatography-mass spectrometry (LC-MS) were greatly outnumbered by gas chromatographic methods. Nevertheless determination of NCS by LC-MS seems to have a promising future in routine analysis. This technique has an exquisite analytical performance in steroid bioanalysis, wide range applicability and provides sensitive and specific results [2]. Beside all the benefits, LC-MS method development has many hinderers. Open, hands-on LC-MS systems are more available when compared to closed, automated platforms. Thereafter, method development, optimization and validation still represent fundamental prerequisites for the application of these methods in research and clinical practice [9] [10] [11].

NCS bioanalysis implies analysing the complex mixtures of low concentration-, structurally similar, and chemically instable steroid structures, and thus represents a specific problem in terms of analyte isolation, identification and quantification [12] [13]. One major problem in LC-MS analysis of NCS in human serum is certainly the matrix effect that can impair greatly the method sensitivity [2]. Considering that a growing variety of compounds is being analysed by LC-MS in clinical laboratories, solving these problems and it is usually up to the analyst to find the appropriate solution. Consequently, large variations in the results among laboratories can be attributed tothe methodological issues [10] [14]. All of the above illustrates the need for specific in-house validation of the NCS bioanalytical methods.

The aim of this study was firstly to develop and optimize reliable methods for quantification of NCS in serum and HDL fraction isolated from serum (HDLNCS). Next objective was to comprehensively investigate the problem of calibration curve construction due to the extensive matrix effect. Finally, our goal was to validate both methods and apply them for measuring total NCS and HDL-NCS concentrations in serum samples obtained from healthy individuals.

## Materials and Methods

### Reagents, samples and instrumentation

Peaks of desmosterol (purity ≥84%), 7-dehydrocholesterol (purity ≥95), lathosterol (purity >99%), campesterol (purity ∼65%) and b-sitosterol (purity TraceCERT® grade) were identified by comparison with corresponding HPLC grade analytical standards (Supelco, Bellefonte, PA, USA). Deuterated internal standard (IS) d6-cholesterol (HPLC grade) purchased from Sigma-Aldrich (St. Louis, MO, USA) was used. Cholesterol standard of ≥99% purity was obtained from Sigma Aldrich (St. Louis, MO, USA). KOH was purchased from POCH (Center Valley, PA, USA), and ethanol, methanol, n-hexane and acetonitrile (HPLC grade) from Fisher (Pittsburgh, PA, USA). Bovine serum albumin (BSA) was obtained from Sigma-Aldrich (St. Louis, MO, USA).

Commercial cholesterol HDL precipitating reagent produced by BioSystems (Costa Brava, Barcelona, Spain) contained phosphotungstate (0.4 mmol/L) and magnesium chloride (20 mmol/L).

Human serum samples were obtained from healthy volunteers after the general medical examination with permission from local Ethical committee and following the guidelines defined by the Declaration of Helsinki. Samples were used for method development and clinical verification. All participants signed an informed consent form before the enrolment.

HPLC method development and sample analyses were done using C-18 Porochell 120-EC column (150×4.6 mm×2.7 μm) (Agilent Technologies, USA).

### Labware preparation

Prior to all sample preparation, glassware were prepared and used in concordance with our previously described protocol. Plastic consumables use was strictly limited to high-density plastics [15].

### Sample preparation for HPLC-MS/MS analysis of serum NCSs' concentration

Firstly, 50 μL of IS (d6-cholesterol, 1 mg/mL) was added into conical reaction tube and dried under the gentle stream of nitrogen. Then, 100 μL of serum was transferred into the same conical glass reaction tube. After brief vortexing, 1 mL of 2% KOH in ethanol was added and vortexed vigorously for 15s to ensure precipitation of proteins. Next, basic hydrolysis was performed by incubating the mixture for 30 min at 45 °C. After cooling down to room-temperature, samples were diluted with 500 μL of deionized HPLCgrade water, and 2 mL of n-hexane was added. The sample was vigorously vortexed for 30s and after centrifuging for 5 min at 1500 x g the top layer was carefully removed and transferred into another clean glass tube. The extraction process was done for three times in total. All the organic layers were joined together. Afterwards, 4 mL of HPLC-grade deionized water was added to wash the excess KOH. Extract was then carefully collected and transferred into clean reaction tube, dried under the gentle nitrogen stream, and reconstituted in 20 μL of HPLC-grade methanol. In the end, 10 μL of methanolic extract was injected into the column. After analysis the concentrations were calculated from the appropriate calibration curves and corrected for the concentration of the sample by evaporation (Cf). Cf for total NCS analysis was 5.

### Sample preparation for HPLC-MS/MS analysis of HDL NCSs' concentration

The protocol for non-HDL particles precipitation was described by the manufacturer of the precipitation reagent. In short, 200 μL of serum was added to 500 μL of precipitation reagent, vortexed thoroughly and left to incubate at room temperature for 10 minutes. Afterwards, the mixture was centrifuged at 6000 rpm for 10 minutes. Supernatant containing HDL fraction of serum (650 μL) was carefully removed and transferred into a glass conical tube containing previously dried IS (50 μL of d6-cholesterol, 1 mg/mL). Afterwards, the abovementioned sample preparation procedure encompassing saponification, extraction, washing, drying and reconstituting in 20 μL of methanol prior analysis was applied. Afterwards, the concentrations were calculated from the appropriate calibration curves and corrected for both the dilution of the sample by the precipitation reagent and concentration of the sample by evaporation (Cf). Cf for HDL-NCS analysis was 32.5.

### Instrumental conditions for HPLC-MS/MS NCSs' analyses

All the analyses were performed under the same chromatographic and MS/MS conditions. Separation of the sterols and oxysterols was achieved by using Porochell 120 EC column (150×4.6 mm×2.7 μm) by Agilent Technologies (USA). Chromatographic conditions included isocratic elution with constant mobile phase flow of 0.6 mL/min and column temperature of 30 °C. The mobile phase was composed of acetonitrile: methanol: water with 0.1% formic acid (80: 18: 2, v/v). Sample injection volume was 10 μL, and all the non-cholesterol sterols were eluted in 45 min. The m/z transitions for each analyte are given in Table 1. Quantification was done using multiple-reaction-monitoring (MRM) on triple quad mass spectrometer Agilent 6420 equipped with APCI ion source. The source conditions were as follows: gas temperature of 325 °C, vaporizer temperature of 250 °C, gas flow of 5 L/min, nebulizer pressure of 30 psi, positive capillary voltage of 2000 V, positive corona current of 4 μA, and positive charging of 2000 V.

**Table 1:** Retention times and MS/MS conditions for internal standard and NCSs

Component	Retention time, min	MRM transitions (m/z)	Collision energy (eV)
Cholesterol-d6 (IS)	30.437	375.3→105.2/95.2/81.3	50/35/52
Desmosterol	20.329	367.2→95.3/81.2	52/52
7-dehydrocholesterol	23.668	367.3→131.3/105.3/81.2	40/50/50
Lathosterol	29.495	369.4→107/95/81.4	40//40/47
Campesterol	36.444	383.2→161/135.3/81.3	20/20/40
β-sitosterol	43.469	397.3→95/69.4/107	40/40/40

### Method validation

After optimizing the quantitation method the appropriate calibration curves were constructed. The relation between the sterol/ IS peak area ratios and the concentration of each sterol was represented by the calibration curves. Fresh calibration standards of 7 different concentration levels were prepared and assayed using the optimized method [16]. LOQ and LOD values were determined by diluting the final extract of low concentration samples and analysing signal to noise ratios (S/N). For LOD target S/N value was 3, whereas for LOD target S/N value was 10. The samples were analysed in pentaplicate for determination of each analytical limit.

Intra-run precision was determined from lowand high-concentration serum pools which were divided in 5 aliquotes each. All five aliquotes were prepared in the same batch and run in triplicate in the same day. This procedure was done for both total and HDL-NCS analysis. Inter-run precision was determined from the same low-and high-concentration serum pools which were divided in 5 aliquotes each. One aliquot was prepared daily for five consecutive days and run in triplicate. The same procedure was done for both total and HDL-NCS analysis.

Recovery studies were performed from serum pool spiked with solutions of 5 different concentrations. The pool was obtained from 20 healthy volunteers. Blank serum pool sample was regarded as the level-zero.

### Clinical evaluation

For the method clinical evaluation, we tested 20 plasma samples from healthy individuals. Inclusion criteria were adult age and absence of any acute or chronic disease, or use of any therapy which could affect lipid status.

Average serum NCS and HDL-NCS concentrations were determined.

### Statistical analyses

Regression analysis was used for standard curve generation, surrogate matrix assessment and the Recovery test. Student t-test was used for comparing the matrices. All data were analysed using IBM ® SPSS® Statistics version 22 software.

## Results

### Method optimization -chromatographic separation, »cholesterol matrix effect« and optimization of the calibration curves

Majority of analytes were successfully separated based on their m/z ratio by triple quadrupole mass spectrometer. Additional chromatographic separation was needed in order to separate isobaric peaks of desmosterol and 7-dehydrocholesterol (both having the same m/z of 367) as well as lathosterol from the dominant cholesterol (both having m/z ratio of 369). Firstly, we tried separating the target NCSs with Porochell 120 EC (75×4.6 mm×2.7 μm) column, but lathosterol and cholesterol could not be separated. Afterwards we managed to separate all the sterols with two-fold longer column Porochell 120 EC (150×4.6 mm×2.7 μm) and aforementioned mobile phase conditions. The resolution factors for desmosterol and 7-dehydrocholesterol was 36, while the resolution factor for lathosterol and cholesterol was 1.6.

**Figure 1:**
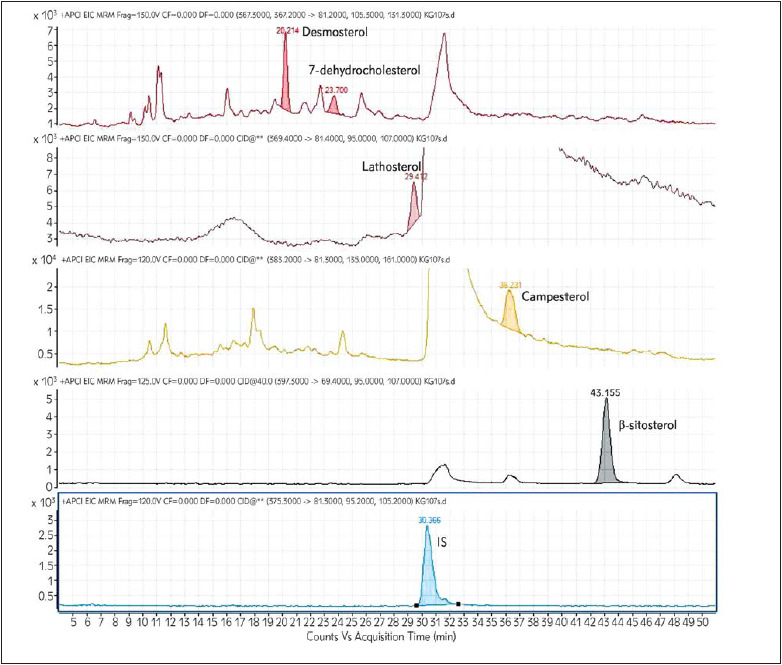
Chromatogram representing all the analytes

The chromatogram of the analysed sample is given in the Figure 1.

Matrix effect was determined after comparing the peak areas of methanolic standard solution of all five sterols with those of the post-extraction samples spiked with the standards at the same concentration levels, and correction for the sample blank. The ionisation enhancement of around 30% was observed for lathosterol, campesterol and β-sitosterol.

Preliminary validation showed the unsatisfactory results of the recovery studies (data not shown), indicating that accurate quantification cannot be done by using methanolic solutions for constructing the calibration curves. Hence, in order to screen for the appropriate surrogate matrix we made methanolic mixture containing all five NCS. This mixture was further divided into five equal aliquots. These aliquots were dried under nitrogen and used to test different matrices. First tested matrix was methanol (MTH); the second was 0.1% bovine serum albumin (BSA); the third was cholesterol (CHL; 5 mmol/L); the fourth had both proteins and cholesterol (BSA+CHL; 0.1%+5 mmol/L). The matrices comparisons were done by analysing NCS area/IS area ratios in different matrices with simple regression analysis test, whereas the significances for b (slope) values were examined with simple t test. The results are shown in Table 2. As seen in the table there was significant difference between CHL and MTH, CHL and BSA, BSA+CHL and MTH, as well as BSA+CHL and BSA, while the difference between BSA and MTH and BSA+CHL and CHL were not prominent. Based on these observations, we concluded that CHL was the best candidate for surrogate matrix, while the BSA solely or in combination with CHL did not add significant value to the matrix effect.

**Table 2:** Slope values for different matrices comparison by simple linear regression Significant differences are represented in bold.Significance for slope values was assessed using Student t-test.The significance level was P 0.05.

Matrix	Cholesterol (5 mmol/L)	BSA (0.1%)	BSA+cholesterol (0.1% + 5 mmol/L)
Methanol	1.600	0.983	1.583
Cholesterol (5 mmol/L)	–	1.630	0.991
BSA (0.1%)	–	–	1.614

Afterwards, it was necessary to determine the appropriate concentration of the cholesterol for the calibration curves construction. Firstly, methanolic solutions of different cholesterol concentrations were prepared (2.5 mmol/L, 3.6 mmol/L, 5 mmol/L, 6.125 mmol/L, 7.5 mmol/L, 10.0 mmol/L). The constant amount of NCS standards was dissolved in each of these cholesterol solutions and the results were again tested using simple regression analysis.

As seen in Table 3, 2.5 mmol/L solution significantly differed from all the other tested solutions, while there was no significant difference between 3.625 mmol/L solution and 5 mmol/L methanolic solution, we opted for 5 mmol/L solution in order to stay close to the expected concentration range of cholesterol in human samples, and mimic the cholesterol matrix effect. However, although 5 mmol/L CHL calibration curve was used to quantitate NCSs which elute from the column after cholesterol (lathosterol, campesterol and b-sitosterol), MTH curve was used for quantitation of desmosterol and 7-dehydrocholesterol, since these compounds were eluted from the column before dominant cholesterol and were not affected by the cholesterol matrix effect. The selection of the appropriate surrogate matrix was confirmed with the results of the recovery study.

**Table 3:** Different cholesterol concentrations comparison Significant differences are represented in bold.Significance for slope values was assessed using Student t-test.The significance level was P 0.05.

	Slope, b(%)				
Cholesterol concentration (mmol/L)	3.625	5	6.125	7.5	10
2.5	1.946	2.063	2.343	2.396	2.915
3.625	–	1.058	1.204	1.231	1.492
5	–	–	1.136	1.162	1.414
6.125	–	–	–	1.023	1.239
7.5	–	–	–	–	1.211

Accordingly, NCS analysis in HDL fraction was performed with MTH curve for desmosterol and 7dehydrocholesterol quantification, whereas lathosterol, campesterol and β-sitosterol were quantified by using 2.5 mmol/L CHL calibration curve.

### Method validation

Standard curves equations for serum NCS and HDL-NCS were given in Table 4.

**Table 4:** Calibration curves, concentration ranges of calibration curves, sterols concentration in healthy subjects, limits of detection (LOD) and quantitation (LOQ) for NCSs *Values for concentrations in real samples, given in the table, are previously corrected for the appropriate concentration / dilution factor (Cf). Cf for total NCS analysis is 5; Cf for HDL-NCS analysis is 32.5.

Analyte	Calibration curve equation andcorrelation coefficient		Calibration curve range (μmol/L)	LOD (μmol/L)*		LOQ (μmol/L)*		Concentrations in healthy sub- jects (N=20) *	
	Total NCS	HDL-NCS		Total NCS	HDL- NCS	Total NCS	HDL- NCS	Total NCS (μmol/L)	HDL-NCS (μmol/L)
Desmosterol	y = 0.051x + 0.0028 r = 0.9996	y = 0.051x + 0.0028 r = 0.9996	5.00–72.34	0.06	0.009	0.20	0.031	3.16 (2.65–4.10)	0.22 (0.17–0.28)
7-dehydro cholesterol	y = 0.030x–0.0148 r = 0.9971	y = 0.030x – 0.0148 r = 0.9971	5.56–37.96	0.07	0.011	0.22	0.034	2.06 (1.18–2.52)	0.31 (0.26–0.36)
Lathosterol	y = 0.012x + 0.0193 r = 0.9993	y = 0.015x + 0.0075 r = 0.9998	3.56–530.19	0.40	0.061	1.40	0.215	23.88 (16.28–33.28)	0.91 (0.67–1.49)
Campesterol	y = 0.050x + 0.0809 r = 0.9968	y = 0.052x + 0.0279 r = 0.9987	3.06–62.39	0.20	0.031	1.20	0.185	5.64 (3.69–6.95)	0.52 (0.43–0.65)
β-sitosterol	y = 0.018x + 0.0088 r = 0.9994	y = 0.020x + 0.0309 r = 0.9982	8.41–62.69	0.10	0.015	0.33	0.051	6.55 (3.89–8.45)	0.91 (0.65–1.15)

According to Food and Drug Administration (FDA) guidelines, the acceptable intra-run and interrun variations were considered to be less than 15% [16]. Both variabilities were satisfactory for each of the sterols'-analysis. These results are summarized in Table 5.

**Table 5:** Intra- and inter run precision for serum and HDL-NCSs level CV – Coefficient of variation

Sterol	Total NCS							
	Inter-run				Intra-run			
	Low		High		Low		High	
	Concentration	CV, %	Concentration	CV, %	Concentration	CV, %	Concentration	CV, %
Desmosterol	2.67±0.159	6.0	4.28 ± 0.227	5.3	2.54±0.186	7.3	4.61 ± 0.425	9.2
7-dehydrocholesterol	1.15±0.093	8.1	2.62 ± 0.207	7.9	1.05±0.083	7.9	2.45 ± 0.156	6.4
Lathosterol	16.20±1.463	9.0	30.80 ± 2.367	7.7	16.23±1.68	10.3	30.96 ± 2.908	9.4
Campesterol	3.66±0.349	9.5	6.24 ± 0.575	9.2	3.41±0.345	10.1	6.04 ± 0.621	10.3
β-sitosterol	3.63±0.168	4.6	8.63 ± 0.421	4.8	3.45±0.163	4.7	8.47 ± 0,532	6.3
Sterol	HDL-NCS							
	Inter-run				Intra-run			
	Low		High		Low		High	
	Concentration	CV, %	Concentration	CV, %	Concentration	CV, %	Concentration	CV, %
Desmosterol	0.15±0.006	4.0	0.25± 0.019	7.8	0.17±0.012	7.1	0.23 ± 0.029	12.6
7-dehydrocholesterol	0.19±0.009	4.5	0.36 ± 0.036	9.8	0.14±0.008	5.8	0.33 ± 0.045	13.6
Lathosterol	0.53±0.020	3.8	1.46 ± 0.093	8.8	0.49±0.018	3.7	1.29 ± 0.108	8.4
Campesterol	0.37±0.019	5.1	0.61 ± 0.050	8.4	0.41±0.036	8.8	0.51 ± 0.060	11.7
β-sitosterol	0.62±0.022	3.5	1.23 ± 0.033	2.5	0.58±0.021	3.6	1.33 ± 0.127	9.6

Satisfactory results were obtained for the difference between the expected concentrations and found concentrations over the five-level concentration range. For the established method, recovery was between 85.3-113.0%, while the acceptable values are in the ±25% range according to FDA guidelines [16]. There was also a good correlation between spike concentrations and found concentrations for each sterol in both sample types. Results of recovery analysis are shown in Table 6.

**Table 6:** Results of the Recovery test for all five sterols

Sterol	Total			HDL fraction		
	Correlation coefficient (r)	Slope (b), %	Spike concentration range (μmol/L)	Correlation coefficient (r)	Slope (b), %	Spike concentration range (μmol/L)
Desmosterol	0.997	113.1	1.08–8.68	0.998	95.8	0.14–2.26
7-dehydrocholesterol	0.998	108.2	0.59–9.49	0.993	95.6	0.07–1.19
Lathosterol	0.999	97.4	1.67–13.25	0.995	88.3	0.21–3.31
Campesterol	0.999	90.7	1.95–15.60	0.996	85.3	0.24–3.90
β-sitosterol	0.994	89.8	1.26–20.25	0.982	87.5	0.12–1.96

### Clinical application

The method was applied to real serum samples from 20 healthy volunteers. Average concentrations are shown in Table 4.

## Discussion

NCS determination provides valuable information on cholesterol biosynthesis and absorption and many of the related metabolic pathways. However, despite their undoubtedly promising clinical potential [17] [18], there is a great variability regarding almost all analytical aspects. Methodological differences significantly contribute to the overall variations in NCS concentrations, compromising the possibilities for extensive meta-analyses. It is believed that up to 25% of variations in the concentration of phytosterols originate from methodological differences [19]. In addition, NCS bioanalysis lacks standardized sample preparation protocols, as well as quantification methods in order to obtain precise and accurate results. Dias et al. particularly indicates the need for validation, pre-analytical, analytical and post analytical variations and overall method standardization for implementing targeted metabolomics-based biomarker analysis in clinical laboratory practice 10. Additionally, conclusions of the first international survey of cholesterol and NCS analysis by chromatographic methods proved there are »the surprisingly high variations in cholesterol and NCS concentrations obtained from analytical assays based on chromatographic separation« and that there is an urge for the harmonization of cholesterol and NCS analysis in serum and plasma [13].

During the method development, we performed preliminary validation in terms of recovery assessment, and acquired unsatisfactory results for lathosterol, campesterol and b-sitosterol. Unsatisfactory results of the recovery studies can be attributed to the inadequate extraction procedure or considerable matrix effect. Although, the usage of internal standard for calibration curve construction serves to account for the extraction and ionisation yields, it is necessary to additionally evaluate the matrix effect on ionisation. Huang et al. [20] propose that the matrix effects assessment should be done in combination with the recovery study in order to examine the overall process efficiency. According to FDA guidelines for bioanalytical method validation, the acceptance criteria for the extraction recovery are not precisely defined. Instead, it is required for the recovery to be 'consistent, precise and reproducible' [16]. Therefore, the extraction recovery, accompanied with the absolute matrix effect should be performed as part of method validation, even if it may not be necessary to establish the method validity [20]. In our particular case, the obtained high values for lathosterol, campesterol and b-sitosterol recovery were in concordance with the observed ionization enhancement, indicating that the problem was not the extraction procedure itself. Additionally, our previous experience during the development of GC/FID method for quantification of the same analytes, by using the similar extraction procedure and having satisfactory extraction yields for all NCS [15], assured us that our further efforts should be focused on resolving of matrix effects, rather than the extraction yield itself.

Indeed, mass spectrometry-based methods in particular may suffer from a specific interference due to the ionization efficiency, which can be hindered by various factors including mobile phase, or sample composition and properties [20]. Firstly, it can be difficult to obtain an appropriate analyte-free medium for analysis of endogenous compounds in biological matrix [21] [22] [23] [24]. Standard-addition method is sometimes a satisfactory alternative if analyte-free calibration material is not available [22]. Nonetheless, it is known that this quantification method causes the loss of sensitivity [24]. Although it appears that matrix effects cannot be completely avoided during LC-MS analysis, there is a constant need for developing optimal analytical conditions that assure the reliable quantification. We decided to use in-house prepared surrogate matrix, containing compounds that were abundantly present in the human serum, in order to satisfy the analytical needs for NCS quantification in both serum and HDL-fraction and provide reliable results that could be used in clinical studies. We opted to test cholesterol, proteins, and cholesterol+proteins solutions [25]. The rationale behind this decision was the fact that cholesterol is highly abundant in the NCS lipid extract used for quantification. The concentration of cholesterol is up to 1000-fold higher in serum compared to the NCSs. This represents a challenge in chromatographic separation of the target NCS, but may also be an obstacle for optimal quantification [26]. Beside cholesterol, we considered that any eventual proteins leftover can be a potential cause of inadequate recovery performance. Hence, we examined BSA as possible surrogate matrix during our method development. We prepared 0.1% BSA methanolic solution and performed a preliminary experiment. BSA did not improve the recovery performance when used as surrogate matrix, whereas BSA+CHL could possibly be used as a surrogate according to the initial comparison with other matrices. In addition, sole CHL methanolic solution showed significant improvement for the lathosterol, campesterol and β-sitosterol. Finally, CHL was chosen over the BSA+CHL mixture due to its unarguably dominant contribution to the matrix effect, reduced complexity and lower possibility to build up both in the column and the instrument. Further on, we needed to adjust the concentration of the CHL solution to be used for this purpose. For this reason we prepared multiple dilutions of CHL methanolic solution, in order to cover the physiological and pathological concentration expected in human serum. The optimal results were achieved with 5 mmol/L CHL solution for the serum NCS analysis. Since the cholesterol is far less abundant in HDL fraction, cholesterol concentration for calibration curve construction was reduced to 2.5 mmol/L. This was also confirmed by testing the different cholesterol solution concentrations. A signif-icant difference was obtained for the use of 2.5 mmol/L versus 3.6 mmol/L solution, while the difference was not significant between 3.6 mmol/L and 5 mmol/L solutions. As proven by earlier studies and confirmed by ours, high cholesterol-to-sterol concentration ratio represents a specific interference in sterol and oxysterol analysis. Namely, quantification of lathosterol, campesterol and β-sitosterol, whose peaks are in the close proximity to cholesterol, was greatly influenced by cholesterol, while desmosterol and 7dehydrocholesterol were not affected by cholesterol due to optimal run duration and sufficient cholesterol elution time. In this study we confirmed that the matrix effect is greatly influenced by the presence and concentration of cholesterol in the serum extracts. Accordingly, this problem needs to be addressed during the construction of the calibration curves and the calibration matrix should be adjusted according to the expected cholesterol concentrations in the analysed samples. Separation of some NCS peaks from dominant cholesterol peak can represent a great challenge. Separation of some NCS peaks from dominant cholesterol peak can represent a great challenge. Similar chromatographic behavior due to similar structure-based properties is observed between cholesterol and lathosterol, since these analytes are isobars and both their peaks have parent mass-to charge ratio of 369 m/z. An adequate runtime provided by appropriate column length and temperature, as well as mobile phase flow, allowed us to successfully separate these two analytes.

Different analytical and research needs, variety of available sample preparation techniques, various separation methods and conditions, and lack of reference analytical method and materials, currently leaves up to the analysts the choice of the most appropriate and the most applicable methodology for their own laboratory. However, this can lead to constant mismatch between different laboratories and their test results. Currently, the only available method check-up for most laboratories is the internal validation of the in-house analytical protocols and the consistency in their application [2]. Our method was fully validated according to the current ICH/FDA bioanalytical guidelines. All validation parameters reached satisfactory values for determination of both serum and HDL NCS in serum.

To the best of our knowledge, this is the first study that examined the methodological aspect of quantifying the aforementioned NCS in HDL fraction, and explored and optimized quantification protocol for both analyses (serum NCS, and HDL-NCS). Method validation was verified in serum samples obtained from healthy subjects and average NCS concentrations were determined in serum and HDL fraction. Determination of NCS in HDL fraction could serve as a future biomarker which can provide information on cholesterol efflux. Namely, even if there are methods dealing with cholesterol and sterol efflux which are based on radiolabelling of the cholesterol and NCS, and in vitro experiments [27], we sought to develop a method that would be more practical in terms of application in routine and clinical studies, and provide additional information on the interplay between cholesterol and lipoprotein metabolism. Wang et al have hypothesized that ABCG family transporters are involved in the cellular excretion of cholesterol and other sterols in a cell-and tissue-specific fashion.They showed that ABCG1 and ABCG4 promote efflux of cholesterol, desmosterol, and possibly other biosynthetic precursors of cholesterol to the HDL particle in the brain [8]. Karuna et al. [28] have already used the similar methodology to ours for estimation of 27-hydroxycholesterol (27OHC) concentrations in HDL fraction and have drawn important conclusions on the causative-consequence relationship between HDL and plasma 27OHC concentrations in several HDL-affecting conditions. Nevertheless, as far as we know this is the first method that considers the analytical aspects of cholesterol synthesis and absorption markers measurement in HDL fraction. However, future chemometric analysis would be valuable addition to fully evaluate this method robustness. Moreover, its clinical potential should be proven in different pathologies.

## Conclusion

The current study describes a newly developed and optimized method for analysis of serum NCS in HDL fraction by LC-MS/MS. Also, the study introduces a comprehensive approach to resolution of the matrix effect in an affordable way by employing cholesterol solution as surrogate medium for serum NCS and HDL-NCS quantification. Adjustment of the quantification method according to cholesterol levels in the samples was proven to ensure more reliable analytical results. Both methods (for serum NCS and HDL-NCS) are successfully validated. Further on, NCS and HDL-NCS were determined in 20 healthy subjects thus confirming the applicability of these procedures for analysis of human samples.

*Funding*: This study was financially supported by a grant from the Ministry of Education, Science and Technological Development, Republic of Serbia (Project No. 175035).

## Conflict of interest statement

The authors state that they have no conflicts of interest regarding the publication of this article.

## List of abbreviations

NCS, non-cholesterol sterol; HDL, high-density lipoproteins; LCMS, liquid chromatography – mass spectrometry; HDL-NCS, non cholesterol sterols in serum HDL fraction; FDA, Food and Drug Administration; ICH, International Council for Harmonization of Technical Requirements for Pharmaceuticals for Human Use; HPLC-MS/MS, high performance-liquid chromatography, triple quad mass spectrometry; apoAI, apolipoprotein A1; ABCA, ATP-binding cassette transporter A; ABCG, ATPbinding cassette transporter G; IS, internal standard; KOH, Potassium hydroxide; LLE, liquid-liquid extraction; LOD, limit of detection; LOQ, limit of quantitation; BSA, Bovine serum albumin; MRM, multiple reaction monitoring; SPE, solid phase extraction; ACN, acetonitrile; MeOH; MTH, methanol; MMI, multi mode ionisation; APCI, atmosphere pressure chemical ionisation; CHL, cholesterol; ESI, electro-spray ionisation; GC/FIDgas chromatography/flame ionization detection; 27OHC, 27-hydroxycholesterol.
